# Sharing errors with human and non-human agents

**DOI:** 10.1093/cercor/bhaf315

**Published:** 2025-12-09

**Authors:** Margherita Adelaide Musco, Lucia Maria Sacheli, Danilo Leggio, Gianpaolo Basso, Eraldo Paulesu

**Affiliations:** Department of Psychology and Milan Center for Neuroscience, University of Milano-Bicocca, Piazza dell'Ateneo Nuovo 1, 20126, Milan (MI), Italy; Department of Psychology and Milan Center for Neuroscience, University of Milano-Bicocca, Piazza dell'Ateneo Nuovo 1, 20126, Milan (MI), Italy; IRCCS Istituto Ortopedico Galeazzi, Via C. Belgioioso 173, 20157, Milan (MI), Italy; Department of Psychology and Milan Center for Neuroscience, University of Milano-Bicocca, Piazza dell'Ateneo Nuovo 1, 20126, Milan (MI), Italy; Department of Neuroscience, Fondazione IRCCS San Gerardo dei Tintori, Via G.B. Pergolesi 33, 20900, Monza (MB), Italy; School of Medicine and Surgery, University of Milano-Bicocca, Via Cadore 48, 20900, Monza (MB), Italy; Department of Psychology and Milan Center for Neuroscience, University of Milano-Bicocca, Piazza dell'Ateneo Nuovo 1, 20126, Milan (MI), Italy; IRCCS Istituto Ortopedico Galeazzi, Via C. Belgioioso 173, 20157, Milan (MI), Italy

**Keywords:** errors, fMRI, joint action, shared goals, social monitoring

## Abstract

Interpersonal action monitoring, i.e., the ability to monitor other people’s actions, is essential during face-to-face interactions. Previous evidence from two independent research lines suggests that both how we represent the interaction goal and the human/non-human nature of the co-actor may affect how we process (and react to) their mistakes. Here, we examined in a full-factorial design whether these two factors modulate how we monitor someone else’s errors during minimally joint tasks. During functional magnetic resonance imaging (fMRI), participants interacted with a human or the computer while sharing or not the goal of playing a melody together (shared vs. individual goal conditions). We used implied-motion pictures of a human hand to represent the human partner’s responses, while a robotic piston represented the computer’s ones. Despite the minimal nature of the interaction, multivariate pattern analysis revealed that it was possible to decode the human/non-human nature of the partner from post-error brain activation patterns but only in the shared goal condition. With both partners, post-error behavioral adaptations in this condition were associated with activation of the pre-supplementary motor area and right anterior insula, brain regions responsible for proactive action control. Goal sharing is thus a powerful factor in boosting interpersonal action monitoring with both human and non-human partners.

## Introduction

One fundamental ingredient of face-to-face interactions is “interpersonal action monitoring,” i.e., the ability to monitor others’ behavioral responses in real time. This capacity allows us to detect others’ errors and, when necessary, adapt our own behavior accordingly, for example, by compensating for their mistakes. Interpersonal action monitoring is essential for successful joint actions, when individuals coordinate their actions to achieve a shared goal (Sebanz et al. 2006) because it guarantees online mutual adjustments: Where one agent fails, the other one can step in and intervene.

An experimental approach to studying interpersonal action monitoring involves examining behavioral and neural responses to others’ errors because errors can only be detected if the co-actor’s behavior is monitored as the interaction unfolds (Vidal et al. 2020). A commonly used behavioral index of interpersonal action monitoring is the observation-related post-error slowing (oPES), that is, a slowdown in reaction times (RTs) following an observed error (Schuch and Tipper 2007; Weller et al. 2018; Musco et al. 2023). The oPES resembles the slowdown that typically occurs after one’s own error execution (Rabbitt 1966). Although there is evidence of a partial dissociation between the two indexes (Picton et al. 2012; Ceccarini and Castiello 2019), it is plausible that the two effects share some neurocognitive resources (for a thorough discussion, see Musco et al. 2023). It has been argued that the oPES reveals the participants’ implicit tendency to correct the partner’s error and produce the action that the partner should have done, thus revealing a mechanism that may contribute to performance optimization in realistic settings (Sacheli et al. 2021, 2022).

A systematic review by Musco et al. (2023) suggests that the representation of the goal(s) underlying an interaction—particularly how relevant the partner’s error is to the individual’s own goal—may modulate post-error responses, at least at the behavioral level. Specifically, the oPES tends to be stronger when observed errors critically impact the observer’s ability to achieve their own or the interaction goal (Castellar et al. 2011; Sacheli et al. 2021), such as when the setter’s error hinders the hitter’s smash in a volleyball match. In other words, experimental evidence indicates that the way individuals represent the goal of a social exchange influences behavioral markers of interpersonal action monitoring by shaping the perceived relevance of others’ errors.

As for the neural correlates of the oPES and their possible modulation, evidence from functional magnetic resonance imaging (fMRI) studies remains limited and inconclusive. For example, De Bruijn et al. (de Bruijn et al. 2009) found that observing a partner’s error activates the posterior medial frontal cortex and the bilateral anterior insula (AI)—an effect consistently reported across studies, as confirmed by the meta-analysis in Musco et al. (2023). However, these activations do not differ between cooperative and competitive contexts, suggesting that contextual social goals may not modulate early neural responses to observed errors.

In addition to this, it has been suggested that, during human–human interactions, the monitoring of others’ actions and errors may rely on a direct matching between one’s own and others’ actions, which could be supported by internal motor simulation processes (see Musco et al. 2023 for a review). Indeed, the observer’s motor repertoire and expertise, which shape the capability of simulating the observed actions, also modulate the detection of observed errors with specific neural patterns (Aglioti et al. 2008; Proverbio et al. 2012; Candidi et al. 2014; Meyer et al. 2016; Panasiti et al. 2016; Pedullà et al. 2020). Here, we refer to direct matching as the putative sensorimotor mechanism by which observed actions are mapped onto the observer’s own motor repertoire, enabling predictive error processing. The potential role of direct matching mechanisms in error monitoring raises the question of whether the same neural mechanisms can support error monitoring in interactions with non-human agents. Indeed, if interpersonal action monitoring depends on such sensorimotor matching, it may be impaired—or even fail entirely—when this matching is not possible, as in the case of interactions with artificial agents lacking human-like morphology or motor primitives (Kilner et al. 2003; Press 2011). This would imply that monitoring others’ actions in human–machine interactions must rely on qualitatively different cognitive processes.

Overall, previous literature suggests that both the nature of the interaction (eg presence/absence of a shared goal) and of the co-actor (human/non-human) may modulate neurocognitive processes underlying interpersonal action monitoring. Nevertheless, the role of these two factors has never been explored in neuroimaging studies within the same full-factorial design so that the neural mechanisms underlying the interplay between goal representation and action simulation in error monitoring remain poorly understood.

In two previous behavioral experiments, we showed that, at the behavioral level, interpersonal action monitoring was enhanced when the interaction was guided by a shared (rather than an individual) goal, both in human–human and human–machine settings (Musco et al. 2025). In the present fMRI study, we build on these findings by investigating the neural mechanisms underlying interpersonal action monitoring. Using a full-factorial design, we test the impact of two key factors—the nature of the interaction (eg presence/absence of a shared goal) and of the co-actor (human/non-human)—on the neural activity associated with error observation during interaction.

**Fig. 1:**
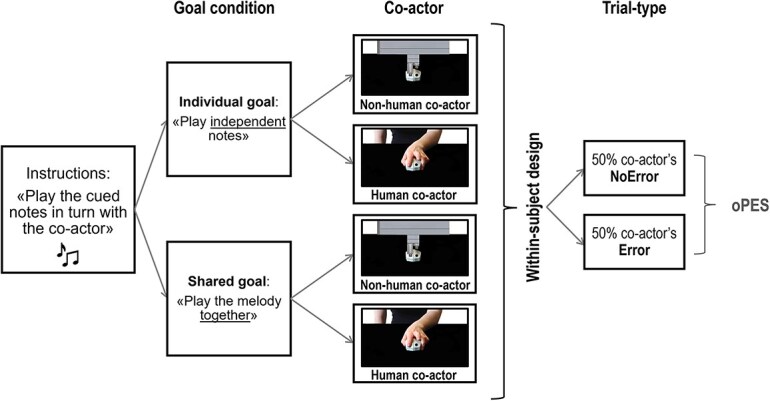
The experiment. The experimental design included the within-subject factors Goal condition, Co-actor, and Trial-type. The task required participants to play the musical notes cued at the beginning of each trial in turn-taking with the (Human and Non-Human) co-actor, during two Goal conditions. In the Individual goal condition, the participant and the co-actor had individual, independent goals, so that the co-actor’s contribution was irrelevant to gaining points. In the Shared goal condition, they shared the common goal of reproducing the cued melody together by playing one note each. In this case, a co-actor's error prevented them from gaining the point. In 50% of the trials of each of the four blocks (Individual goal—Human, Individual goal—Non-Human, Shared goal—Human, Shared goal—Non-Human), the co-actor played the wrong note (Error vs. NoError trials).

We adopted a task that proved to be effective in exploring the neural correlates of behavioral adaptations to observed errors during minimally joint motor interactions (Sacheli et al. 2022). Participants took turns with a co-actor—presented onscreen—in performing previously learned sequences of two notes or single notes. The co-actor was described as either another human participant (actually, a confederate of the experimenters) or a computer. Importantly, all stimuli and responses were generated by the same software script, regardless of the co-actor’s identity. To preserve the minimalistic nature of the interaction, the only differences between the two co-actor conditions were their visual appearance and the cover story provided to participants. The task took place under two experimental conditions that manipulated goal representations. In the Shared goal condition, the co-actors’ performance was necessary for gaining points, while in the Individual goal condition, their behavior was irrelevant to the participants. Please note that the term “shared goal” here refers to the minimal definition provided by Butterfill (2012), who suggests that an interaction goal can be defined as such when it “coordinate[s] multiple agents’ goal-directed activities around an outcome to be achieved as a common effect of their efforts” (Butterfill 2012, p. 37). In all trials, the co-actor initiated the sequence and committed an error 50% of the time (Error trials), allowing us to assess behavioral and neural responses to observed errors. Importantly, in the Individual goal condition, a cue specified the expected, yet irrelevant, co-actor’s behavior, whereby their performance could be deciphered as a hit or an error. See Fig. 1 and Videos S1 and S2 for a representation of the paradigm and trial timeline.

At the behavioral level, and in line with previous findings (Musco et al. 2025), we expected that the oPES would be modulated solely by goal representation: Specifically, greater slowing was anticipated in the Shared goal condition compared to the Individual goal condition, irrespective of the co-actor’s nature. Regarding the neural correlates of such an effect, a prior study using a similar motor interaction paradigm found that observing a human partner’s error elicited activation in a distributed fronto-parietal and fronto-opercular network, including medial frontal regions and the AI (Sacheli et al. 2022), typically associated with performance monitoring (Ullsperger et al. 2014). Notably, the strength of these activations correlated with the magnitude of the oPES. In the present study, we expected to replicate this finding and explored how the nature of the interaction (presence/absence of a shared goal, Shared vs. Individual goal condition) and of the co-actor (Human vs. Non-Human co-actor) may modulate these effects. If direct matching mechanisms play a major role and interpersonal action monitoring mainly relies on motor simulation processes, post-error neural responses should differ depending on whether the co-actor is perceived as human or non-human (main effect of the co-actor’s nature on post-error brain responses). Alternatively, the role of goal representation processes would be revealed by post-error neural activity being primarily modulated by the relevance of the co-actor’s actions for the participant’s goals (main effect of goal condition, individual vs. shared). Importantly, we also explored whether these two mechanisms are inter- or independent, by testing for a significant interaction between co-actor’s nature and goal condition.

## Materials and methods

### Behavioral procedures

#### Sample size estimation

The sample size was established through a simulation-based power analysis for mixed models (Kumle et al. 2021) and was based on the effect that we wanted to replicate from the previous fMRI study with a similar paradigm, that is, the significant difference between RTs from the Error and NoError trials in the joint action task (Sacheli et al. 2022). The raw data that we used for power analysis can be found in the Supplementary Material of Sacheli et al. (2021), where the behavioral data of the fMRI study were discussed as a replication experiment. We chose the smallest effect size of interest (Lakens et al. 2018) of 60% of the effect found in the previous study, and the critical *t*-value was set at 2. Power estimates were obtained by simulating and then modeling 10,000 independent experiments using the mixedpower package in R (Kumle et al. 2018), with increasing numbers of participants, with the aim of selecting the sample size corresponding to a power estimate of at least 0.80. The power analysis indicated that with *n* = 24, the power estimate was 0.80.

#### Participants

Twenty-six healthy volunteers responded to a call on the local Sona System (https://milano-bicocca.sona-systems.com/) and were tested. Two participants were excluded from the analyses (see below), so the final sample included 24 participants (11 males, 13 females, age range 19 to 31, mean age 23.25 ± 3.02 years). All participants were native Italian speakers, right-handed (Edinburgh Handedness Inventory (Oldfield 1971), mean score 0.86 ± 0.12), had a normal or corrected-to-normal vision, reported an absence of neurological or psychiatric disorders, were naive about the purposes of the experiment, signed written informed consent in line with the ethical standards of the 1964 Declaration of Helsinki and later amendments, and were debriefed on the purposes of the study at the end of the experimental procedures. The protocol was approved by the University of Milano-Bicocca ethics committee (protocol number: 735).

#### Stimuli and apparatus

**Fig. 2:**
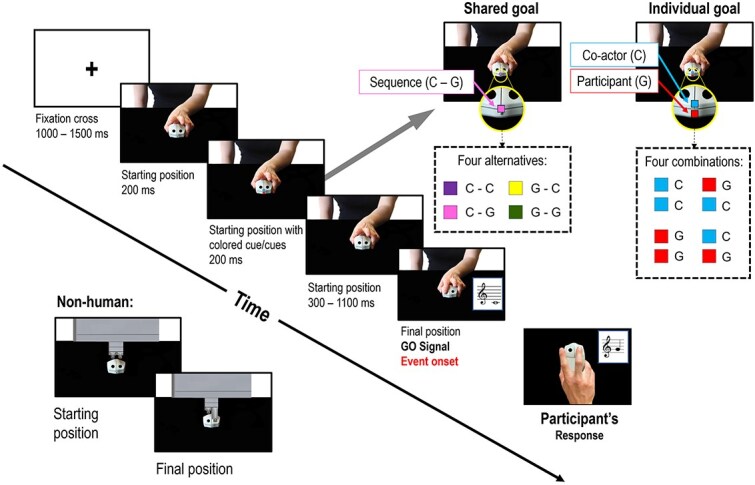
Trial timeline of a NoError, Human co-actor trial. The trial timeline was identical in all experimental conditions. The stimuli used in the Human co-actor condition depicted frontally a human left hand grasping a computer mouse similar to the participant’s one: The “starting position” picture represented the index and middle finger of the co-actor lifted over the computer mouse, and the “final position” picture depicted the end of the button-press action. Instructions specifying the melodies/single notes that participants had to perform were delivered by superimposing small colored squares over the center of the co-actor’s computer mouse in the starting position picture. The images of the Non-Human co-actor (bottom left) showed an artificial agent that covered the same screen area as the human co-actor. Instead of the fingers, the Non-Human co-actor used pistons to press the buttons, reaching the buttons from above and lacking any resemblance with human fingers flexing over the mouse. The depicted table and mouse were identical in shape, color, and position. The white background covered almost the same percentage of pixels (10.56% of white pixels for the human pictures and 9.15% for the non-human ones). The Individual goal and Shared goal conditions differed only for the cues (shown on the top-right corner): In the Individual goal condition, two cues indicated the co-actor’s and participant’s independent notes to be played, whereas in the Shared goal condition, a single cue triggered the representation of the whole two-note melody. The onset of the final position picture was used as the onset in the event-related fMRI analysis.

During the fMRI session, the participants saw visual stimuli through a mirror placed over the head coil reflecting an MRI-compatible screen placed behind the scanner (40″; resolution: 3840 × 2160). A response box placed under their right hand (Resonance Technology Inc., Northridge, CA, USA) recorded the participants’ responses. Pressing a button with the index finger generated a C note (∼261 Hz), whereas pressing a button with the middle finger generated a G note (∼392 Hz). The notes had the same duration (100 ms). Auditory stimuli were provided through MRI-compatible headphones. Stimuli presentation was controlled by E-Prime3 software (Psychology Software Tools, Inc.). The visual stimuli consisted of three different types of images for each co-actor, which were presented in sequence within each trial and created the impression of a movement (see Fig. 2 and Video S2).

#### Experimental procedure

The fMRI task was an adaptation of the task adopted by Sacheli et al. (2022). The experiment conformed to a 2 × 2 × 2 within-subject factorial design with Goal condition (Shared goal vs. Individual goal), Co-actor (Human vs. Non-Human), and Trial-type (NoError vs. Error) as factors.

The task required participants to play (i) two sequential notes (“melodies,” Shared goal) or (ii) single notes (Individual goal) in turn-taking with a co-actor (Human/Non-Human) whose responses were presented on the screen. Colored cues on the screen signaled what melody or note the participant had to play in each trial, according to a previously learned association between colors and instructions (see Supplementary Material for details on the learning session). In the Shared goal condition, a single cue indicated what melody they had to play together; in the Individual goal condition, the participants saw two colored squares, indicating separately their own and their co-actor’s notes (Fig. 2). For this reason, participants knew which note the co-actor was about to play in both contexts, at variance with our previous studies (Sacheli et al. 2021, 2022), thus enabling them to encode the co-actor’s action as a hit or an error in both Goal conditions. Participants had to wait until the co-actor played their note before responding. The final result was always a two-note melody among the following: GG, GC, CG, and CC. The instructions led participants to perform a G/C note 50% of the time, and the combination of the participant’s and co-actor’s actions was physically congruent or incongruent in 50% of the trials. See Videos S1 and S2.

Upon their arrival at the MRI unit, each participant met a confederate, who was introduced as their “human” partner. Before entering the scanner, the participant and the confederate underwent a training session in which they were required to play the previously learned two-note melodies (Shared goal) or single notes (Individual goal) in turn-taking.

Participants underwent four experimental fMRI runs (Shared goal—Human, Shared goal—Non-Human, Individual goal—Human, Individual goal—Non-Human, counterbalanced order). In both Goal conditions, in the Human blocks, participants were told that the co-actor’s responses were controlled by the other participant (actually, the confederate). In the Non-Human blocks, they were told that the responses were controlled by the computer, which was programmed to make errors sometimes. Participants were told that a general ranking would be compiled based on the points accumulated in the four tasks. The ranking was only communicated to the participants by the end of the data collection. In half of the trials, the co-actor played the wrong note (Error vs. NoError trials): While in the Shared goal session, this error led to losing one point, in the Individual goal session, it was irrelevant to the participant.

The trial timeline was identical in the four runs (Fig. 2 and Supplementary Material). Overall, participants performed 48 trials per experimental conditions (384 trials overall). Before starting each run, the participants performed three practice mini-blocks (12 NoError trials) to familiarize themselves with the task.

#### Post-fMRI behavioral procedures

Once outside the scanner, the participants performed a control task in which they saw the color cues from the Shared goal condition five times each and had to play the associated two-note melody. This task was used to determine whether the participants remembered the association between colors and melodies at the end of the experiment. All participants reached the accuracy threshold of 80%. The participants also answered some debriefing questions reporting their subjective experiences. Finally, they were asked how much, during the experiment, they actually believed that the other participant (i.e., the confederate) was controlling the displayed co-actor’s responses in the Human condition. They responded through a 100 mm vertical Visual Analogue Scale (Hayes and Patterson 1921) ranging from “Not at all” (0 mm) to “I fully believed” (100 mm).

### Behavioral data analyses

We measured accuracy, i.e., the proportion of correct responses, and RTs, i.e., the time delays between the go-signals and the participant’s button presses. Out of the 26 recruited participants, we excluded 2 participants (final *n* = 24) because they showed a grand-mean accuracy that fell below the group median value of more than 1.5 times the interquartile distance (Tukey 1977). All participants included in the analyses showed an RT grand mean that fell within 2.5 SD above/below the group mean RTs. Only trials in which the participants provided the correct response were considered in the RT analyses.

The behavioral data were analyzed in jamovi 2.3.26 (The jamovi project 2022). To investigate post-error behavioral adaptations, we analyzed the oPES, which was computed as follows: oPES = $\frac{RT_{Error}-{RT}_{NoError}}{\left({RT}_{Error}+{RT}_{NoError}\right)/2}$,where ${RT}_{Error}$ and ${RT}_{NoError}$ are, respectively, the mean RTs in the Error and NoError trials. The index is normalized on the mean RTs in order to control for interindividual differences across participants. The index was computed for each participant in each of the four experimental conditions (Shared goal—Human, Shared goal—Non-Human, Individual goal—Human, Individual goal—Non-Human). Data were normally distributed (Shapiro–Wilk test *P*s > 0.05). All tests of significance were based upon an α level of 0.05.

**Fig. 3:**
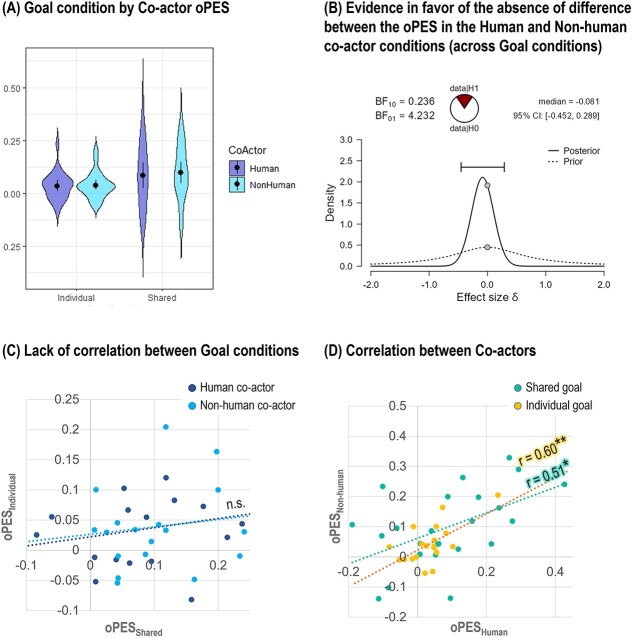
Behavioral results. A) The violin charts plot the oPES distribution in the four experimental conditions (from left to right: Individual goal—Human, individual goal—Non-Human, Shared goal—Human, Shared goal—Non-Human). The oPES was computed with the following formula: oPES = $\frac{RT_{Error}- RT_{NoError}}{\left( RT_{Error}+ RT_{NoError}\right)/2}$, where ${RT}_{Error}$ and ${RT}_{NoError}$ are, respectively, the mean RTs in the error and NoError trials. The black dots and lines correspond to the group mean and standard deviation. The oPES is significantly greater in the Shared goal than the Individual goal condition. B) The Bayesian paired-sample *t*-tests showed evidence in favor of the null hypothesis, that is, the absence of difference between the participants’ oPES in the Human and Non-Human conditions. Here, we report the plot from the *t*-test that was run on the oPES averaged across the Shared goal and Individual goal conditions. C) The Pearson correlation between the oPES in the Individual goal and Shared goal conditions was not significant, neither in the Human (blue dots) nor the Non-human co-actor condition (light-blue dots). (D) The oPES effect in the Human and Non-human conditions correlated in both goal conditions. ^**^ = *P* < 0.01; ^*^ = *P* < 0.05; n.s. = not significant.

We tested what factor/s modulate the oPES by applying a repeated-measures analysis of variance (ANOVA) having Goal condition and Co-actor as categorical independent factors. We used Pearson correlation tests to explore whether the oPES effects measured in each experimental condition were correlated. As the absence of a significant difference between experimental conditions (eg between the Human and Non-Human co-actor conditions) was one of the expected outcomes, we also planned to apply a Bayesian statistical analysis to assess the strength of evidence in favor of the null hypothesis in such instances. All behavioral results are reported in Fig. 3.

### fMRI data acquisition and analyses

#### Data acquisition

Whole-brain functional T2-weighted images were acquired using a 3.0 T Ingenia CX scanner (Philips S.p.A., Milan, Italy), located in the Fondazione IRCCS San Gerardo dei Tintori, equipped with gradient-echo echo-planar imaging (EPI) (repetition time [TR] 2,000 ms, echo time [TE] 30 ms, 35 transversal slices, descending not interleaved acquisition, 4 mm slice thickness with no interslice gap, FA 75°, FOV 240 mm, matrix size 80 × 80, voxel-size = 3 × 3 × 4 mm). Two hundred twenty volumes per run were acquired for each subject. Each run lasted about 7 min. The first five volumes recorded from each functional run were removed to allow for steady-state tissue magnetization. A high-resolution T1-weighted anatomical scan (TR = 8.1 ms, TE = 3.7 ms, 207 sagittal slices, FOV = 256 mm, matrix size = 256 × 256, FA = 8°, inversion time [TI] = 1,000 ms) was acquired before the fMRI session.

#### Preprocessing

We reconstructed images and then converted raw data from the Digital Imaging and Communications in Medicine (DICOM) to the Neuroimaging Informatics Technology Initiative (NIfTI) format using MRIconvert software (https://neuro.debian.net/debian/extracts/mriconvert/copyright). We adopted MATLAB R2019a (MathWorks) using Statistical Parametric Mapping software (SPM12, Wellcome Department of Imaging Neuroscience, UCL) to perform the preprocessing of the data and all the univariate data analyses. We first realigned and unwarped the fMRI scans to reduce the influence of head movements during the scan session and the effect of magnetic field distortions. The unwarped images were coregistered with the T1-weighted structural image of each participant, which was then segmented and stereotactically normalized into the SPM12 template (tmp.nii) to allow for group analyses of the data. Finally, deformation fields used for T1 segmentation were applied to the coregistered functional scans. We then interpolated the data matrix to produce voxels with 2 × 2 × 2 mm dimensions. The normalized scans were smoothed using a Gaussian filter of 10 × 10 × 10 mm to improve the signal-to-noise ratio and allow for valid application of the family-wise error rate (FWER) correction at the cluster-level in the second-level (i.e., group-level) univariate analyses, as recommended by (Flandin and Friston 2019). For the multivariate pattern analyses (MVPA, see below), a 4 × 4 × 4 mm Gaussian filter was used to preserve local voxel information but still reduce high spatial frequency noise in the images (Gardumi et al. 2016).

The data of each participant were screened using Artifact Detection Tools (ART, Whitfield-Gabrieli, http://www.nitrc.org/projects/artifact_detect) to identify outlier scans in the global signal and movement. We marked time points as outliers if scan-to-scan variations in the global signal overcame 3 SD from the mean and if the compounded measure of movement parameters exceeded 1 mm scan-to-scan movement average. The outlier time points were accounted for by adding regressors of no interest in the single-subject analyses. Outlier volumes per participant were, on average, 2.68% ± 2.92% in the Shared goal—Human condition, 2.38% ± 1.87% in the Shared goal—Non-Human condition, 2.00% ± 1.93% in the Individual goal—Human condition, and 2.69% ± 3.80% in the Individual goal—Non-human condition.

#### First-level analyses (single-subject level)

The Blood Oxygenation Level Dependent (BOLD) signal was first convolved with a canonical hemodynamic response function (Worsley and Friston 1995) and high-pass-filtered (128 s). In the first-level (single-subject) analysis, condition-specific fixed effects were calculated with SPM12, separately for each run. In an event-related design, the onset of the event corresponded to the onset of the partner’s final-position picture (go-signal for the participant, see Fig. 2). In each run (Shared goal—Human, Shared goal—Non-Human, Individual goal—Human, Individual goal—Non-Human), we characterized the BOLD signal associated with the observation of the co-actor’s response separately for NoError and Error trials. Separate regressors modeled experimental confounds, including trials in which the participants provided a wrong response (accuracy = 0) and the realigning parameters calculated in the preprocessing step. For each run (Shared goal-Human, Shared goal—Non-Human, Individual goal—Human, Individual goal—Non-Human), we then calculated the following contrasts: simple effects of NoError trials, simple effects of Error trials, and the contrast effect of Error > NoError. The first-level analysis was run separately for the images smoothed with the 10 × 10 × 10 mm and the 4 × 4 × 4 mm Gaussian filter.

#### Second-level analyses (group level)

The group-level analyses were conducted in three steps:

1. **Full-factorial ANOVA: analysis of Goal condition by Co-actor by Trial-type interactions.** The first fMRI analysis was aimed at characterizing brain activity associated with the observation of a co-actor’s error, and eventually, its modulation by goal representation and direct matching processes. We entered the simple effects of the Error and NoError trials of each of the four runs in a full-factorial ANOVA that conformed to random effect analyses. The ANOVA included Goal condition (Shared goal vs. Individual goal), Co-actor (Human vs. Non-Human), and Trial-type (NoError vs. Error) as within-subject factors.
2. **Regression analyses: brain activations predicted by the oPES effect**. As a second step of the fMRI data analyses, we explored whether the brain activations following an observed error were differently associated with the related behavioral adaptations (i.e., the strength of the oPES effect) depending on the Goal condition. In two voxel-by-voxel linear regression analyses (one for each level of the Goal condition factor), we used as a continuous predictor the strength of the oPES effect, which was entered into the dependent-sample *t*-test including the following contrast images: (i) Human Error > NoError and (ii) Non-Human Error > NoError. The analysis was performed at the whole-brain level and allowed us to investigate what brain activations are predicted by the strength of the oPES effect and the possible interaction with the factor Co-actor.
3. **Multivariate pattern analyses (MVPA): classification of the Co-actor based on the post-error activation patterns**. The hypothesis that a human error is processed differently from an artificial one was tested by using a pattern classification analysis performed with the Pattern Recognition for Neuroimaging Toolbox (PRoNTo, http://www.mlnl.cs.ucl.ac.uk/pronto). MVPA permits testing the hypothesis that information is encoded in a distributed pattern of voxels rather than by the activation difference in individual voxels (Schrouff et al. 2013). The MVPA was run separately for the two Goal conditions on the contrast images obtained from the first-level analysis performed on the volumes smoothed with the 4 × 4 × 4 mm Gaussian filter (Error > NoError, two per subject, one for each co-actor). These contrast images were used as input for the pattern analysis, in which a kernel classifier was trained to identify voxel activation patterns in the contrast images with a support vector machine (SVM) using LIBSVM implementation (Chang and Lin 2011). We restricted the analysis to the brain regions included in the main effect of error observation (brain regions showing a main effect of Error > NoError—FWER-corrected at the cluster level, corresponding to approximately 14,584 voxels). We used a nested cross-validation procedure to train the model and optimize the model hyperparameters. The external loop was used for assessing the model performance: we adopted the leave-one-subject-per-group-out (LOSGO) cross-validation procedure, resulting in 24-fold cross-validation. In each fold, one input image per class (the Human and Non-Human Error > NoError contrast images of a specific participant) was left out from the training set and used as test data. The internal loop was used for optimizing the models’ hyperparameters (soft-margin parameter C): As in the external loop, we used a LOSGO cross-validation scheme. In this vein, test data were neither used for training nor for the optimization of hyperparameters, guaranteeing a noncircular analysis. We mean-centered the features across training data. The significance of the model performance was determined through 5,000 permutations of the training labels.

## Results

### Behavioral data

For the sake of clarity, we report in Table S1 the accuracy group medians and RTs group means for each experimental condition. In the Supplementary Material, we also report the results of the generalized mixed model on the accuracy data and of the linear mixed model on the RTs data. Here, we describe the results of the analyses on the oPES, that is, the effect used in the fMRI regression analyses.

#### Manipulation check

A non-parametric one-sample t-test showed that the VAS scores significantly differed from 0 (median = 54.50, range = 11 to 100, W = 300, *P* < 0.001), suggesting that most participants were moderately convinced by the cover story, i.e., moderately believed that the Human co-actor’s responses were controlled by an actual participant.

#### Goal condition × Co-actor ANOVA

Table 1 shows the oPES group means. The repeated-measures ANOVA showed a significant main effect of Goal condition [*F*(1,23) = 5.40, *P* = 0.029, η^2^_p_ = 0.19]: The oPES was stronger in the Shared (adj mean = 0.09, SE = 0.02) as compared to the Individual goal condition (adj mean = 0.04, SE = 0.01). The main effect of Co-actor and the interaction effect were not significant. Figure 3A.

**Table 1:** The table shows the oPES group means and standard errors of the mean (SEM). The oPES was computed as oPES = $\frac{\boldsymbol{RT_{Error}}-\boldsymbol{RT_{NoError}}}{\left(\boldsymbol{RT_{Error}}+\boldsymbol{RT_{NoError}}\right)/\mathbf{2}}$, where ${\boldsymbol{RT}}_{\boldsymbol{Error}}$ and ${\boldsymbol{RT}}_{\boldsymbol{NoError}}$ are, respectively, the mean RTs in the Error and NoError trials. SEM, standard error of the mean.

	**Shared goal**		**Individual goal**	
	**Human**	**Non-Human**	**Human**	**Non-Human**
**oPES mean**	0.086	0.098	0.035	0.039
**oPES SEM**	0.03	0.03	0.01	0.01

#### Bayesian t-tests

Given the lack of a significant difference between the oPES following a Human and Non-Human error, we applied a Bayesian paired-sample *t*-test to assess the strength of evidence in favor of the null hypothesis (no difference between conditions), separately for each Goal condition and averaged across conditions. The Bayesian Factor (BF10) was lower than 0.3 in the Shared goal condition (BF10 = 0.243), Individual goal condition (BF10 = 0.241), and in the average across goal conditions (BF10 = 0.244). Figure 3B. These results show moderate evidence in favor of the null hypothesis, that is, the absence of difference in the oPES after human and non-human errors.

#### Pearson correlation tests

The oPES correlated between the Human and Non-Human co-actor conditions both in the Shared goal [*r*(22) = 0.510, *P* = 0.011] and Individual goal [*r*(22) = 0.601, *P* = 0.002] conditions. None of the other possible correlations was significant. These results suggest that while the oPES in the Shared and Individual goal conditions may rely on different mechanisms, the same cognitive processes may determine the oPES with the two co-actors (Figure 3C).

### fMRI data analysis

#### Full-factorial ANOVA

**Fig. 4:**
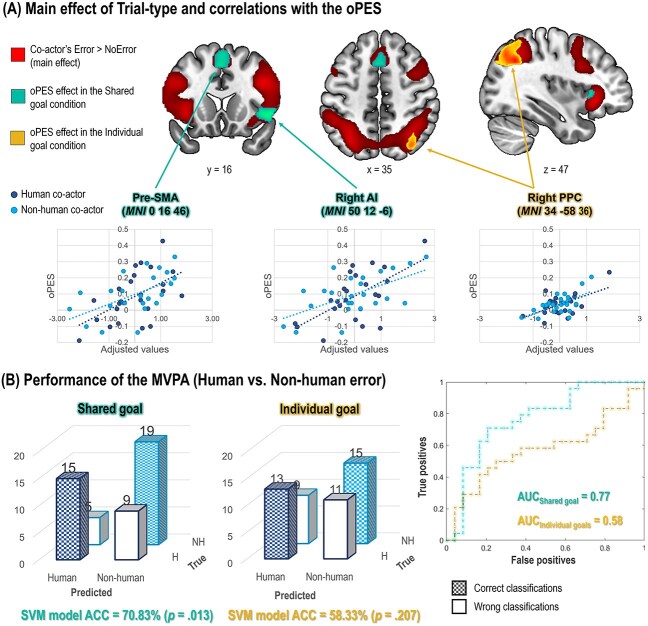
fMRI results. A) In red, the brain activations corresponding to the direct contrast Error > NoError  (i.e., the main effect of Error). In green and yellow, the clusters whose activation was predicted by the magnitude of the oPES in the Shared goal and Individual goal conditions, respectively (i.e., the results of the regression analyses). For visualization purposes, only regions included in the main effect of Error are shown here (see Fig. S2 and Table S4 for the whole-brain results). The oPES was computed as oPES = $\frac{RT_{Error}- RT_{NoError}}{\left( RT_{Error}+ RT_{NoError}\right)/2}$, where ${RT}_{Error}$ and ${RT}_{NoError}$ are, respectively, the mean RTs in the error and NoError trials. Pre-SMA = pre-supplementary motor area; AI = anterior insula; PPC, posterior parietal cortex. (B) Performance of the MVPA. The MVPA was performed in the areas that are significantly more active after a co-actor’s Error as compared to the NoError condition [in red in (A)]. On the left, the confusion matrices represent the proportion of correct and wrong classifications for each category (Human vs. Non-human). On the right, the receiver operator characteristic (ROC) curve plots the true positive rate against the false-positive rate. AUC, area under the ROC curve. The activation maps are visualized at *P*_uncorr_ < 0.001 at the voxel level and *P*_FWE-corr_ < 0.05 at the cluster level.

The whole-brain ANOVA showed a main effect of Trial-type (Error vs. NoError), indicating stronger brain responses to a co-actor’s Error in areas responsible for action monitoring, including the pre-supplementary motor area (pre-SMA), the posterior medial frontal cortex (pMFC), the frontal operculum and AI bilaterally, in addition to bilateral premotor and posterior parietal regions, as well as the left middle temporal gyrus (Table 2 and Fig. 4A). The Goal condition and Co-actor main effects were also significant (Fig. S1, Tables S2 and S3). None of the interaction effects were significant.

**Table 2:** The table shows the significant brain activations in the error > NoError contrast. All the reported effects meet a family-wise error rate (FWER) correction at the cluster level. The regional effects that also survived a voxel-wise FWER correction are marked with (^*^). We report a maximum of 16 coordinates (local maxima) per cluster, each placed at least 4 mm apart, as reported by default in SPM12. *k* = number of voxels in a given cluster. “pMFC” = posterior part of the medial frontal cortex; “pre-SMA” = pre-supplementary motor area. “X,” “Y,” and “Z” refer to MNI stereotaxic coordinates.

	**Left hemisphere**				**Right hemisphere**			
**Brain area (Brodmann area)**	***X***	***Y***	***Z***	***Z*-score**	***X***	***Y***	***Z***	***Z*-score**
	***Posterior medial frontal cluster*** *k* = 825, *P*_FWER-corr_ = 0.005							
pMFC—pre-SMA (6)					0	18	54	5.0^*^
Medial superior frontal gyrus (8)					4	32	42	3.6
Middle cingulum (32)					4	34	38	3.6
	***Left inferior frontal cluster*** *k* = 3,603, *P*_FWER-corr_ < 0.001				***Right inferior frontal cluster*** *k* = 4,388, *P*_FWER-corr_ < 0.001			
Inferior frontal gyrus, pars orbitalis (47)	−40	40	−2	3.9	34	24	−6	6.3^*^
					44	26	−2	5.9^*^
					46	34	−10	5.7^*^
Inferior frontal gyrus, pars triangularis (44)	−48	22	28	5.9^*^				
Inferior frontal gyrus, pars opercularis (44)	−42	10	28	5.9^*^	44	12	30	6.6^*^
	−52	14	6	4.4^*^				
Precentral gyrus (6)	−46	8	46	3.6				
Middle frontal gyrus (6)					38	8	54	4.2
Insula (47)	−36	24	−2	7.0^*^				
	***Bilateral posterior parietal cluster*** *k* = 5078, *P*_FWER-corr_ < 0.001							
Superior parietal lobule (7)					18	−68	60	4.9^*^
					28	−68	54	4.7^*^
Inferior parietal lobule (7/40)	−30	−60	44	6.1^*^	40	−48	48	5.5^*^
	−32	−56	46	6.0^*^				
	−46	−42	46	5.3^*^				
	−42	−44	44	5.2^*^				
Angular gyrus					34	−56	44	5.7^*^
Precuneus (7)	−10	−66	48	3.8	12	−62	48	3.9
	***Left middle temporal cluster*** *k* = 960, *P*_FWER-corr_ = 0.002							
Middle temporal gyrus (21/22)	−60	−52	20	5.2^*^				
	−54	−50	4	4.2				

#### Regression analyses

We explored whether the brain activations following an observed error (represented by the Error > NoError contrasts) were differently associated with post-error behavioral adaptations (i.e., the strength of the oPES) depending on the Goal condition. To this aim, we ran two whole-brain voxel-by-voxel linear regression analyses (one per Goal condition, Shared and Individual) on the Error > NoError contrast images, with the strength of the oPES effect as continuous predictor. Here, we discuss significant results included within the set of brain regions encoding the observed error (i.e., included in the activation map corresponding to the main effect of Error, Fig. 4A). Additional significant activation clusters that emerged from this whole-brain analysis are reported in the Supplementary Material (Fig. S2). In the Individual goal condition, we found an association between the oPES strength and the activity in the right posterior parietal cortex (PPC). Instead, in the Shared goal condition, the analysis showed a parametric association between the oPES effect and the recruitment of pre-SMA and right AI. See Fig. 4A and Table S4. Within the brain regions encoding the observed error (main effect of Error), the regression analysis showed no significant interaction effects with the factor Co-actor.

#### MVPA analyses

The univariate analysis failed to detect a significant interaction effect, suggesting a different brain response following human and non-human errors. We applied a multivariate pattern analysis to explore the possibility that the information might be retained in the overall activation patterns of the brain regions showing a main effect Error > NoError (see the ANOVA). An SVM classifier was able to correctly classify the co-actor’s nature (Human vs. Non-Human) only in the Shared goal condition (total accuracy = 70.8%, *P*_5000perm_ = 0.013) and not in the Individual goal one (total accuracy = 58.3%, *P*_5000perm_ = 0.208). Figure 4B.

## Discussion

The present study sheds light on the neurocognitive mechanisms underlying interpersonal action monitoring by exploring the modulatory role of the two key factors that, according to previous studies, may modulate our ability to face a partner’s mistake. We anticipated two scenarios based on which of these two factors plays a role. On the one hand, if direct matching processes between one’s own motor repertoire and the observed actions play a role in interpersonal action monitoring, the behavioral adjustments following observed errors and the related neural activations would differ depending on the human vs. non-human nature of the co-actor. On the other hand, if how we represent the interaction goal—and, relatedly, the relevance of co-actor’s contribution to our own goals—supports interpersonal action monitoring, its behavioral and neural correlates would be shaped by the goal of the interaction (individual or shared), determining similar post-error responses across different co-actors.

Altogether, as discussed below, our results suggest a prepotent effect of the goal manipulation on the processing of co-actors’ errors. Indeed, although the fronto-parietal and fronto-opercular networks responsible for action monitoring were always engaged in response to an observed error, the error-induced behavioral adaptations were based on the activation of different sub-sets of brain regions depending on the presence/absence of a shared goal guiding the interaction. This was true independently of the co-actor, suggesting that interactions with human and non-human co-actors build on similar neurocognitive bases. Most importantly, our results also indicated that sufficient information was encoded in the patterns of brain activity to discriminate between human and non-human errors, but only if a shared goal was in place.

### Observed errors and expectation violation

The fMRI data showed the existence of a general network activated after observed errors in all experimental conditions. In line with our previous study (Sacheli et al. 2022), this network includes brain regions located in the posterior medial frontal cortex (pMFC), bilateral AI, frontal operculum, bilateral premotor and posterior parietal regions, and left posterior middle temporal gyrus. These results also align with previous studies on action monitoring in humans and non-human primates (Ninomiya et al. 2018; Moreau et al. 2022).

Other authors have found similar inferior frontal and temporo-parietal activations after detecting unexpected sensory information (Downar et al. 2000; Corbetta and Shulman 2002; Vossel et al. 2014; Grundei et al. 2023). Moreover, previous literature showed that error and expectation violation processing share common neural resources, mainly located in the pMFC, inferior frontal gyrus, and AI, both for own (Wessel et al. 2012) and observed actions (Desmet et al. 2014; Schiffer et al. 2014; Cracco et al. 2016). We thus argue that these activations reflect a general signal arising from the action monitoring system when the expectations on the co-actor’s action, induced by the cue, are violated. Although different cues were used in the individual and shared goal conditions, both induced expectations regarding the co-actor’s actions, as shown by the lack of a significant Trial-type (Error vs. NoError) by Goal condition interaction effect. It is worth noting that in our paradigm the error rate was high (50%), but this was not sufficient for overriding expectations induced by the learned association between cues and co-actor’s actions. This result points to the powerful effect of observed errors despite their frequency, in line with the results of previous experiments in which post-error neurophysiological responses were present for error-rates that were even higher (80%) than the one presented here (Pezzetta et al. 2018).

It is also worth noticing that the significant mediofrontal cluster in our results did not extend to the anterior cingulate cortex (ACC), which is often recognized as a crucial hub for error processing. The ACC is known to be the neural source of the error-related negativity (ERN), which is generated by both executed (Gehring et al. 1993; Falkenstein et al. 2000; Debener et al. 2005) and observed errors (Miltner et al. 2004; van Schie et al. 2004). Recent evidence nevertheless suggests that ACC recruitment during error processing may depend on task characteristics (Grabowska et al. 2025), and meta-analytic findings indicate that ACC activation is not consistently recorded during the observation of other people’s errors (Musco et al. 2023). One possible explanation is that, in observational contexts, error detection may rely more strongly on adjacent pMFC regions and on the frontal–insular networks. Future studies that directly compare executed and observed errors while manipulating task types will be essential to clarify the circumstances under which the ACC is engaged in error monitoring.

### Observed errors and shared goals

The overlap of neural resources dedicated to elaborating errors across different goal conditions does not necessarily entail a complete correspondence of the cognitive processes involved. As a matter of fact, the magnitude of the observation-related post-error slowing (oPES) did not correlate across the shared goal and the individual goal conditions, suggesting the two oPES may arise from distinct processes.

There are two lines of evidence in favor of this speculation. First, the oPES was stronger in the shared goal condition, where the co-actor’s error directly affected the possibility of achieving the shared goal. During a joint action (Sebanz et al. 2006), establishing a shared goal leads agents to incorporate the representation of the partner’s expected behavior into a common motor plan (what have been defined as *dyadic motor plan*, see Sacheli et al. 2018), making the partner’s error more relevant to the self. Indeed, instead of being a mere perceptual deviation from expectations, the co-actor mistake prevents the overall shared goal achievement (here, the short melody execution). This may be the reason why post-error reactions are stronger at the behavioral level and shared goal interactions imply more accurate action monitoring overall (Moreau et al. 2022). Within this line, the shared goal condition, independently of the presence of a co-actor’s error, recruited a wider network (see Supplementary Material) including ventral premotor brain regions that proved to be crucial in supporting “dyadic motor plan” representations (Sacheli et al. 2019, 2023).

Second, the oPES measured in the individual goal and the shared goal conditions correlated with the activity of different brain regions. In the individual goal condition, the oPES correlated with the activity of the right posterior parietal cortex (PPC), which has been linked to attentional capture by external and internal stimuli (Mevorach et al. 2006; Hodsoll et al. 2009; Heinen et al. 2011; Giacometti Giordani et al. 2023). Previous studies showed that the inhibition of the right PPC caused a reduction in the interference of a distractor during visual search tasks (Hodsoll et al. 2009). Accordingly, in our study, participants with less activation in the right PPC after a co-actor’s error showed a smaller oPES. On the other hand, in the shared goal condition, the oPES was positively correlated with the activation of the pre-SMA and the right AI, which have been related to intentionality and proactive control of action (Cieslik et al. 2015; Gavazzi et al. 2021; Hung et al. 2018; Xu et al. 2016; R. Zhang et al. 2017). The AI has been implicated not only in the control side of intentionality, but also in its motivational dimension (Brass and Haggard 2010). In the context of joint action, the AI may modulate post-error adaptations by encoding the affective significance of a partner’s error, as suggested by (Koban and Pourtois 2014). When the partner’s contribution is essential for the shared goal achievement, errors may be appraised with a negative affective valence, which could represent the key mechanism driving post-error adjustments (see below the discussion on the adaptive orienting theory of error processing).

Finally, we ought to mention that our results align with previous studies showing that prior knowledge about an observed movement weigh more than the co-actor’s perceptual features in modulating the recruitment of motor simulation processes (Hortensius and Cross 2018). This is one of the reasons why the fronto-parietal network responsible for motor simulation of others’ actions is also active when people observe artificial agents’ actions (Gazzola et al. 2007; Cross et al. 2012), at least under certain conditions (Press 2011; Kupferberg et al. 2018; Miyamoto et al. 2023). We add here that the *relevance* of the partner’s action to the observer’s goal may also have a significant role. As a matter of fact, the brain network that is significantly more active during the shared goal than individual goal conditions includes areas within such a fronto-parietal network, independently of the agent’s appearance.

### Observed errors and the co-actor’s nature

While goal relevance primarily shaped post-error adaptations, the question remains whether the human/non-human nature of the co-actor also leaves a trace in neural processing. Our multivariate pattern analyses (MVPAs) showed that participants’ brains discriminated between errors made by human and artificial co-actors, but only in the shared goal condition. Because MVPAs were run on contrast images encoding the difference between error and correct actions, this effect cannot be explained by perceptual differences. By contrast, the only univariate significant effect of human > non-human partners emerged in the main effect contrast (not controlled for perceptual differences), with two significant clusters in the left and right middle temporal gyrus, a region linked to biological motion processing (Grossman et al. 2000).

Taken together, these results suggest that the co-actor’s nature is encoded in post-error neurofunctional patterns only when it can potentially affect the interaction unfolding, i.e., within a shared goal framework. In our paradigm, such neural sensitivity did not translate into differences in post-error behavioral adaptation. This pattern is in line with most previous work showing comparable post-error adjustments for human and non-human agents (Castellar et al. 2011; Huberth et al. 2019; Somon et al. 2019; Neszmélyi and Pfister 2025; Pesci et al. 2025), despite some inconsistencies (Desmet et al. 2014; Pfister et al. 2020). Importantly, however, the fact that the system encodes a partner’s nature in the context of errors may still be relevant in real-life interactions, potentially influencing behavioral measures beyond the oPES or shaping the emotional dynamics of joint performance.

Overall, our findings indicate that the crucial factor for successful adaptation is not the agent’s nature but the presence of a shared goal, which triggers proactive motor-control mechanisms (Sacheli et al. 2021, 2022; Musco et al. 2025). This has direct implications for human–robot collaboration: emphasizing shared goals may foster the formation of “dyadic” motor plans (Sacheli et al. 2018), thereby enabling reciprocal adaptations between humans and collaborative robots equipped with intention-reading capacities (Ehrlich and Cheng 2018; Zhang and Doyle 2023). The more accurate the dyadic motor plan the human agent forms about the interaction, the more predictable the interaction will be and the more precise the reciprocal adaptation, leading to safer and optimal interactions. We thus suggest that future research on human–robot interaction should shift the focus from optimizing single agents to optimizing the interactive context itself, with shared goal representations as a key leverage point.

### The adaptive orienting theory of error processing

The results described above are in line with an application to the observation domain of the “adaptive orienting theory of error processing,” which postulates different stages in the elaboration of one’s own executed error (Wessel 2018). According to this model, the violation of expectations regarding action-action or action–outcome associations first activates an “automatic cascade” of steps, i.e., the inhibition of ongoing motor and cognitive activity and the attentional orienting to identify the violation source, which is then followed by controlled processes finalized to the adoption of adaptive measures leading, in the absence of time constraints, to increased accuracy (Wessel 2018; Choo et al. 2023). These late, controlled processes are error-specific and finalized to improve the general performance. With the present work, we demonstrate for the first time that the error-processing cascade from Wessel’s model can be applied to the domain of others’ error processing, as explained below.

In our study, participants showed an oPES in both the individual and shared goal conditions. This replicates previous results showing that observing a co-actor’s error causes a performance impairment (Musco et al. 2023) and is coherent with the idea that deviations from the expectations are indeed detected by our cognitive system, even when the error does not impact the observer’s goals. The behavioral results are corroborated by the neurofunctional ones, which show that the brain activation elicited by error observation is not modulated by the goal condition and overlaps with areas associated with expectation violation processing.

For what regards the second part of the cascade, we argue that its implementation will depend on how relevant to the observer the compensation for the co-actor’s error is. According to the adaptive orienting theory of error processing (Wessel 2018), behavioral adaptation following the initial mismatch signal varies based on the necessity to adjust subsequent actions. Previous evidence shows that only if a shared goal is present, a co-actor’s error can induce in the participants the implementation of remedial actions that would guarantee (in an ecological context) the achievement of the shared goal, regardless of the deviation from the original plan (Sacheli et al. 2021, 2022; Musco et al. 2025). Indeed, the oPES is greater in the shared goal condition, where the co-actor’s error prevents the shared goal achievement, and it does not correlate with the oPES measured in the individual goal condition. At the same time, the evaluation of possible remedial actions may include a deeper encoding of the co-actors’ features (not only their human/non-human nature but also, for example, whether they are teammates or opponents, as in the study by Denul and colleagues; Denul et al. 2025), which then determines what type of remediation will be selected. In the present study, while post-error neural patterns of activation encoded the co-actor’s nature, no corresponding behavioral effect emerged. This discrepancy may stem from the minimal interactive context we employed, and it opens a crucial avenue for future research on how interaction complexity modulates the link between neural encoding of the partner’s features and behavioral adaptations.

### Conclusion

We showed that the presence of collaborative goals has the potential to override other factors, like the alien nature of the non-human partner, while interacting. While we were able to specify the core neurocognitive system that may sustain interpersonal action monitoring in joint actions, future studies should focus on its modulation due to additional factors. Importantly, future studies should move beyond the present results by studying interpersonal action monitoring in more realistic scenarios, both as concerns the two co-actors and the error rate. Indeed, the experimental manipulation that allowed us to draw a distinction between the co-actor’s human/non-human nature was minimal, being based on a cover story and on minimally distinctive visual features (lacking kinematic cues). Although we adopted a 50% error rate to ensure sufficient trials for statistical power and MVPA classification, we acknowledge that such a high frequency might reduce the probability of believing in the cover story. As a consequence, the visual analogue scale assessing the beliefs in the cover story was much variable, which could be seen as a possible limitation of the study. In a study applying a similar paradigm, the beliefs did not modulate error-related behavioral adaptations, thus making not likely an effect on our results (Musco et al. 2025). Nevertheless, future studies should vary factors related to the artificial co-actor, such as motion characteristics, prior knowledge about its behavior, or anthropomorphism, to test if they modulate the human partner’s behavioral performance. Moreover, it remains to be determined the impact on interpersonal action monitoring of various degrees of reciprocal adaptation and modulation of intentionality attribution (Navare et al. 2024) when interacting with non-human agents. Addressing these issues will be critical not only for advancing models of interpersonal action monitoring but also for informing the design of collaborative robots capable of flexible and adaptive interaction with humans.

## Supplementary Material

Musco_CerebralCortex_SupplementaryMaterials_bhaf315

Video_S1_bhaf315

Video_S2_bhaf315

## Data Availability

All the behavioral and fMRI data are publicly available in the OSF framework at the following link: https://osf.io/62xaw/.
