# Supplementary material for: Sharing errors with human and non-human agents
Source: Cereb Cortex. 2025 Dec 9;35(12):bhaf315. doi: 10.1093/cercor/bhaf315 (PMC12687879; doi:10.1093/cercor/bhaf315)
Supplement: Musco_CerebralCortex_SupplementaryMaterials_bhaf315 [file musco_cerebralcortex_supplementarymaterials_bhaf315.docx]

**Supplementary Material for**

Sharing Errors with Human and Non-Human Agents

Margherita Adelaide Musco*, Lucia Maria Sacheli*, Danilo Leggio, Gianpaolo Basso, Eraldo Paulesu

*Corresponding authors.

Email: margherita.musco@unimib.it; lucia.sacheli@unimib.it

**This PDF file includes:**

Supplementary Text

Supplementary References

Figure S1 to S2

Tables S1 to S4

**Other supporting materials for this manuscript include the following:**

Videos S1-S2

# Supplementary Text

## Materials and Methods

**Learning session.** The fMRI session was preceded by a learning session, which could be performed no earlier than six days before the experimental session, in which the participants were familiarized with the task. During the learning session, the participants learned: (i) the association between finger presses and the ensuing two possible notes, and (ii) the association between the color cues provided as instruction and the musical notes (“two-notes melody” in the Shared goal condition, “single notes” in the Individual goal condition) to be played during the fMRI experiment. The learning session (about 30 min) was divided into two parts (proper learning and recognition parts): Both parts were first performed in one Goal condition and then repeated in the other one (the order of the Shared goal/Individual goal conditions was counterbalanced between the participants). By design, we planned to test only participants who successfully completed both parts of the learning phase (accuracy threshold equal to 80%). All the participants included in the sample passed the threshold.

First, the participants were required to repeat single notes to learn the association between buttons and notes. Subsequently, the participants learned the association between colors and two-notes melodies (Shared goal condition) or single notes (Individual goal condition) separately for the two Goal conditions. There were two possible associations between colors and sequences / single notes (counterbalanced between participants). The participants first heard each sequence of notes (Shared goal condition) or single note (Individual goal condition) while being concurrently presented with the corresponding color cue. They were asked to reproduce it immediately. Each musical sequence was shown five consecutive times. Afterward, participants were randomly presented with the color cues and required to produce the corresponding musical sequence. The color cue corresponding to each musical sequence was presented ten times. Then, participants performed a recognition task: They heard each musical sequence eight times (random presentation) and had to choose the corresponding color cue. At last, participants were presented with the instructions for the task they were to perform during fMRI and had a brief training session to learn the task (12 NoError trials), separately for each of the four blocks (Shared goal-Human, Shared goal-Non-Human, Individual goal-Human, Individual goal-Non-Human). Participants were told that the training was a simulation of the actual task and that the co-actor’s response was thus simulated in the Human condition.

**Number of trials and trial timeline.** In each trial, the participants watched (and heard) the co-actor playing a note and then responded by pressing one of the two buttons on the mouse. Each fMRI run (Individual goal-Human, Individual goal-Non-Human, Shared goal-Human, Shared goal-Non-Human) included 96 trials, organized into six mini-blocks: Each mini-block included four trials, namely the four sequences/pairs of notes, presented in a randomized order. In each mini-block, the co-actor played the right note in only two trials (NoError trials) and made an error in the remaining two (Error trials). The six mini-blocks presented different combinations of correct/wrong sequences of/single notes. Each of the six mini-blocks also included a black screen, lasting 3,000 – 4,000 ms (so that the mean duration corresponded to the mean duration of a trial), which served as an implicit baseline for the fMRI analyses.

The trial timeline was identical in the four tasks (Shared goal - Human, Shared goal - Non-Human, Individual goal - Human, Individual goal - Non-Human, see Fig. 2 in the main text). It started with a fixation cross (500 ms), followed by the starting-position picture (200 ms); then, the starting-position picture containing the color-cued instruction appeared (200 ms). Next, the starting-position picture reappeared for a variable stimulus onset asynchrony ranging from 300 to 1100 ms. The co-actor’s final position picture and the co-actor’s note were then presented, and this was the participants’ go signal. The participants were asked to respond as quickly and accurately as possible. After the participant’s response, a black screen (filler) of a variable duration was presented so that the total duration of the trial was about 3,500 ms. This was done to increase the temporal distance between consecutive events of interest (i.e., the onset of the partner’s final position picture and note). See Fig. 2 and Videos S1-S2.

## Behavioral Data Analyses

In the main text, for the sake of brevity, we describe the analysis performed on the oPES, that is, the behavioral index of post-error behavioral adaptations that we used as predictor in the fMRI regression analyses. The oPES data were analyzed in the statistical programming environment jamovi (version 2.3.26, (The jamovi project, 2022)), with the GAMLj (Gallucci, 2019) and JSQ (JASP Team, 2018; Morey & Rouder, 2018; Rouder et al., 2009) modules.

Here, we report the mixed model analyses run on accuracy (ACC) and reaction times (RTs) data to test whether the Goal condition (Shared goal vs. Individual goal) and Co-actor (Human vs. Non-Human) factors modulate the impact of the co-actor’s errors on performance (e.g., lower ACC and higher RTs in Error as compared to No Error trials). To this aim, we ran a generalized logistic mixed model on the ACC data and a linear mixed model on the RTs data, considering as fixed effects the factors Goal condition (Shared goal vs. Individual goal), Co-actor (Human vs. Non-Human), and Trial-type (NoError vs. Error trials) and their interactions. The Satterthwaite method for the degrees of freedom and the Wald method for the confidence intervals were applied. Given the models complexity, which poses problems of convergence and singularity, we applied the complex random intercepts approach to deal with the random effects structure (Scandola & Tidoni, 2024). Complex random intercepts are categorical random slopes converted into random intercepts: they are treated as uncorrelated and follow the assumption of homoskedasticity for the participants with respect to the experimental conditions. Given that the maximal random model (Barr et al., 2013) never converged, we applied the pipeline proposed by Scandola and Tidoni (2024), starting with the “full-CRI” model (including all fixed effects also as random intercepts) and when facing singularity or convergence issues, removing the random intercept with the lower variance, in an iterative way, until we found a non-singular model that converged. We always included by-subjects random intercepts. The mixed models were run in R version 4.3.1, by using the lme4 (Bates et al., 2015), lmerTest (Kuznetsova et al., 2017), performance (Lüdecke et al., 2021), car (Fox & Weisberg, 2018), and emmeans (Lenth, 2024) packages.

## Results

**Behavioral results**

**ACC data.** The ACC was very high (grand-mean ACC = 0.96 ± 0.02, see Table S1). The generalized logistic mixed model on the ACC data^^[[1]](#footnote-1)^^ (R^2^_marginal_ = 0.19, R^2^_conditional_ = 0.40) revealed a significant main effect of Goal condition (χ^2^(1) = 45.29, *p* < .001) and Trial-type (χ^2^(1) = 36.66, *p* < .001), indicating that participants were more accurate in the Individual goal than in the Shared goal condition (mean .995, SE= 0.002, vs. .969, SE = 0.007) and in the NoError than the Error trials (mean .992, SE = 0.002, vs. .981, SE = 0.004). None of the interaction effects were significant.

**RTs data.** In line with the ACC results, the linear mixed model on the RTs data^^[[2]](#footnote-2)^^ (R^2^_marginal_ = 0.06, R^2^_conditional_ = 0.48) revealed a significant main effect of Goal condition (F(1,23) = 31.02, *p* < .001), Co-actor (F(1,22.9) = 6.24, *p* = .020), and Trial-type (F(1,22.9) = 16.87, *p* < .001)), indicating that participants were faster in the Individual goal than in the Shared goal condition, when interacting with the Non-Human co-actor than the Human co-actor, and in the NoError trials as compared to the Error trials. The main effects were further specified by a significant Goal condition × Trial-type interaction (F(1,22.9) = 9.09, *p* = 0.006). None of the other interaction effects were significant.

# Figure S1. Co-actor and Goal condition main effects. In green, the difference in neural activation between the Shared goal and Individual goal conditions. In blue, the brain areas that are more active during interactions with the Human than Non-Human co-actor. The activation maps are visualized at *p_uncorr_* < .001 at the voxel level and *p_FWE-corr_* < .05 at the cluster level. L = left; R = right.
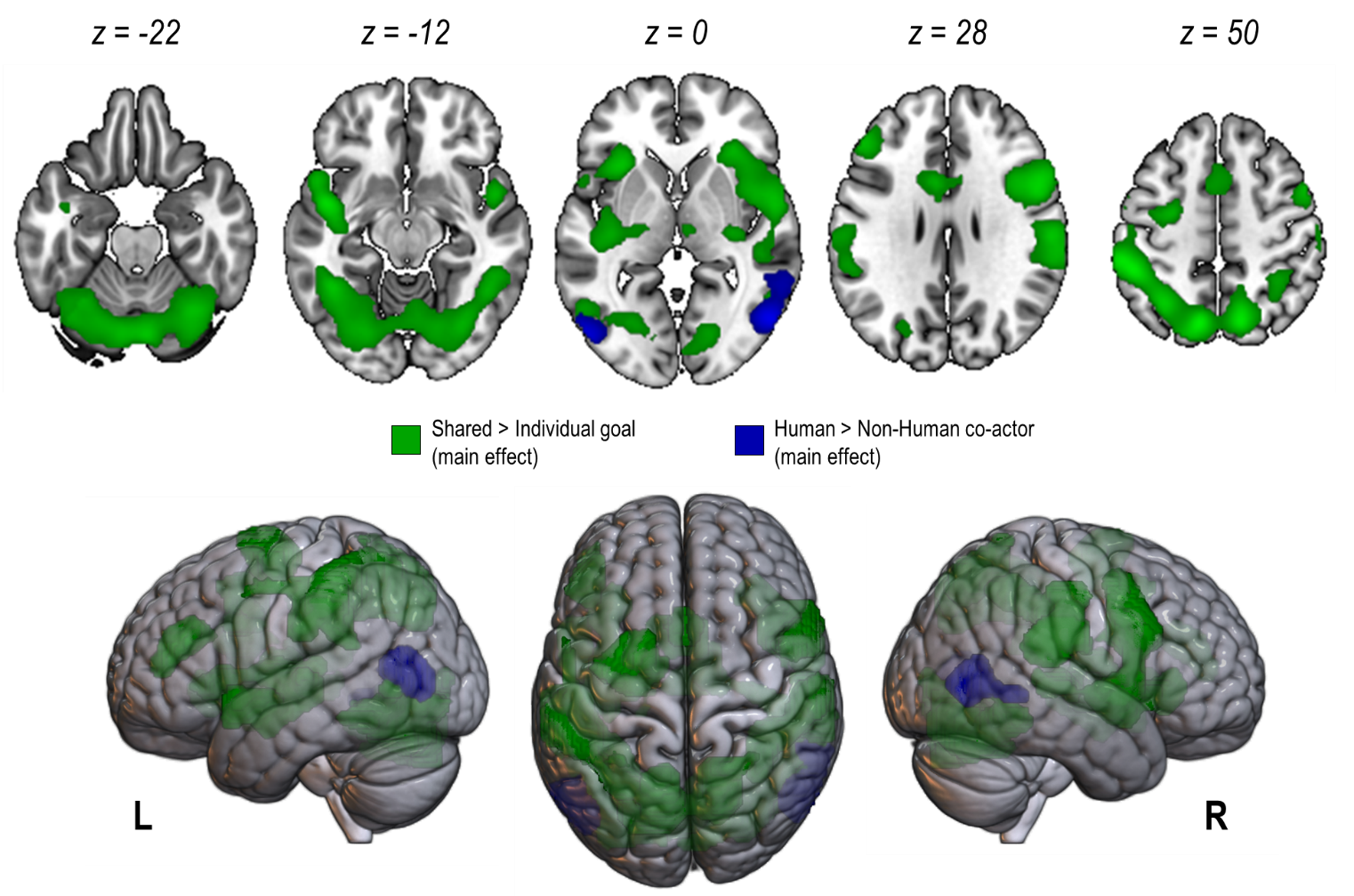


#
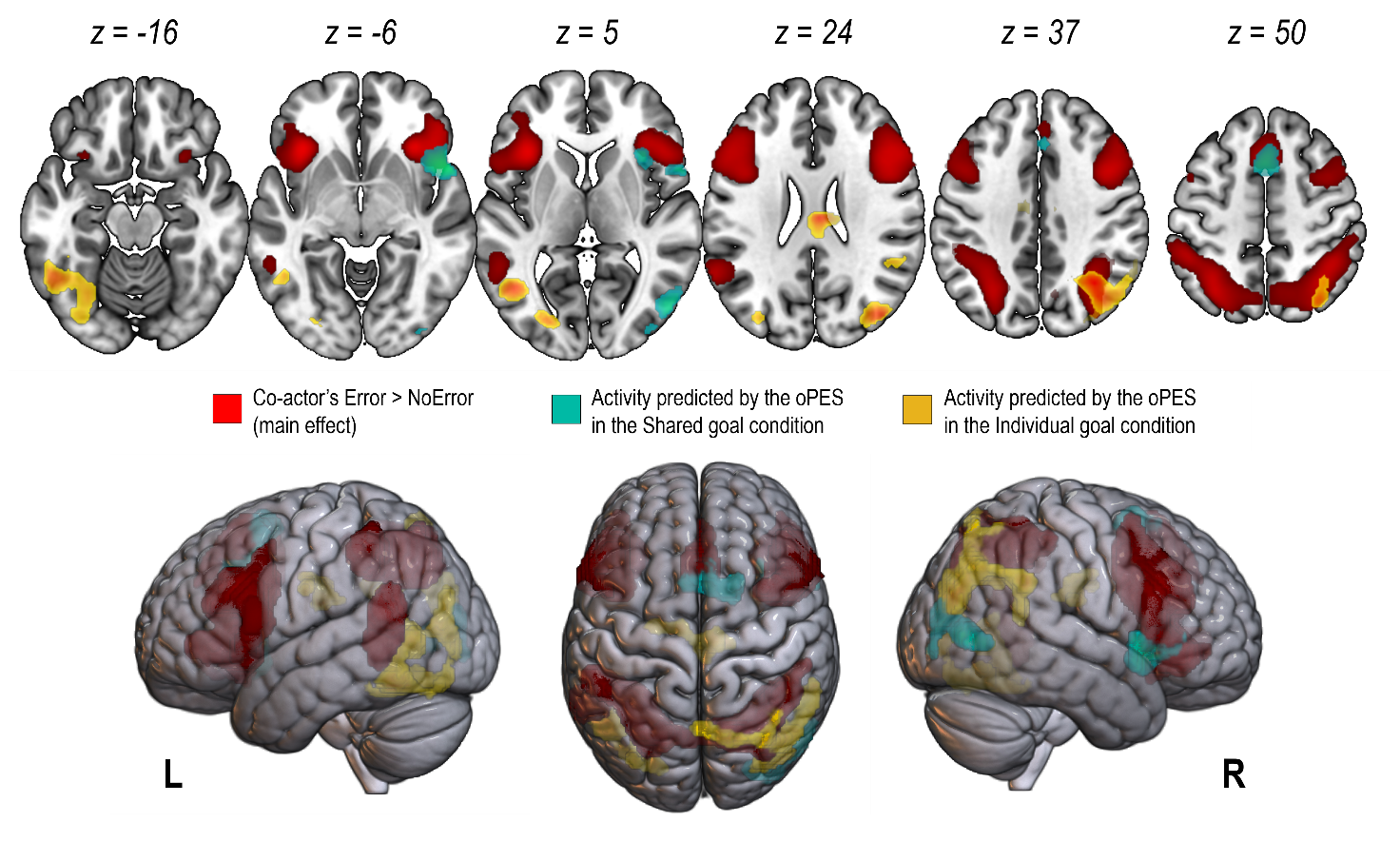
Figure S2. Whole-brain results that emerged from the regression analyses. The figure shows the brain activity predicted by the strength of the oPES in the Shared (light blue) and Individual (yellow) goal conditions, superimposed over the main effect of Error (in red). In addition to the clusters of activation included in the main effect of Error, discussed in the Main Text, in the Shared goal condition, the strength of the oPES also predicted the activity of a cluster in the right occipito-temporal cortex. Instead, in the Individual goal condition, the strength of the oPES predicted the activity of one cluster in the left occipito-temporal cortex and one in the white matter. The activation maps are visualized at *p_uncorr_* < .001 at the voxel level and *p_FWE-corr_* < .05 at the cluster level. L = left; R = right.

# Table S1. Raw accuracy data (ACC, Group Median and Range) and Reaction Times (RTs, Group Mean and Standard Deviation) Values. As concerns the RTs, we calculated the individual mean RTs per each experimental condition, excluding from the calculation the trials in which the participants gave the wrong response, the trials with a reaction time lower than 50 ms, and the outlier values that fell 2.5 SDs above or below each individual's mean for each experimental condition calculated in accurate trials only.

| **ACC** | **Shared goal** | | | | **Individual goal** | | | | |
| --- | --- | --- | --- | --- | --- | --- | --- | --- | --- |
|  | **NoError** | | **Error** | | **NoError** | | | **Error** | |
| **Human** | 0.98  (0.85 - 1) | | 0.93  (0.71 – 0.98) | | 1  (0.92 - 1) | | | 1  (0.77 - 1) | |
| **Non-Human** | 0.98  (0.75 - 1) | | 0.94  (0.77 – 1) | | 1  (0.92 - 1) | | | 1  (0.94 - 1) | |
|  |  |  | |  | |  |  | |  |
| **RTs (ms)** | **Shared goal** | | | | **Individual goal** | | | | |
|  | **NoError** | | **Error** | | **NoError** | | | **Error** | |
| **Human** | 347.64 ± 114.22 | | 382.08 ± 130.55 | | 277.79 ± 79.13 | | | 289.69 ± 89.16 | |
| **Non-Human** | 315.95 ± 109.02 | | 353.47 ± 135.02 | | 267.22 ± 88.58 | | | 278.76 ± 95.53 | |

# Table S2. The table reports the significant brain activations in the Shared > Individual goal contrast (i.e., the main effect of Goal condition emerged from the ANOVA). All the reported effects meet a family-wise error rate (FWER) correction at the cluster level. (*) indicates peaks also surviving an FWER correction at the voxel level. We report a maximum of 16 coordinates (local maxima) per cluster, each placed at least 4 mm apart, as reported by default in SPM12. k = number of voxels in a given cluster. ‘pMFC’ = posterior part of the medial frontal cortex. X’, ‘Y’, and ‘Z’ refer to MNI stereotaxic coordinates.

|  | **Left Hemisphere** | | | | **Right Hemisphere** | | | |
| --- | --- | --- | --- | --- | --- | --- | --- | --- |
| **Brain area (Brodmann area)** | **X** | **Y** | **Z** | **Z-score** | **X** | **Y** | **Z** | **Z-score** |
|  | ***Posterior medial frontal cluster*** k = 845, *p*_FWER-corr_ = .004 | | | | | | | |
| pMFC – pre-SMA and Middle cingulum (24/32) | -8 | 10 | 30 | 4.3 | 0 | 10 | 50 | 3.7 |
|  | -- | -- | -- | -- | 8 | 10 | 30 | 3.6 |
|  |  |  |  |  | 8 | 0 | 32 | 3.5 |
|  | ***Left superior temporal and thalamic cluster*** k = 2406, *p*_FWER-corr_ < .001 | | | |  | | | |
| Insula (47) | -34 | 20 | 2 | 4.9* | -- | -- | -- | -- |
|  | -36 | -2 | 8 | 3.9 | -- | -- | -- | -- |
|  | -38 | -6 | 8 | 3.9 | -- | -- | -- | -- |
|  | -38 | -10 | 6 | 3.9 | -- | -- | -- | -- |
| Superior temporal pole (38) | -50 | 10 | -12 | 4.4* | -- | -- | -- | -- |
| Superior temporal gyrus (21) | -44 | -2 | -12 | 5.2* | -- | -- | -- | -- |
|  | -38 | -20 | -4 | 4.3 | -- | -- | -- | -- |
|  | -48 | -20 | 4 | 3.7 | -- | -- | -- | -- |
| Thalamus | -22 | -16 | -4 | 3.6 | -- | -- | -- | -- |
|  | -16 | -14 | 6 | 3.2 | -- | -- | -- | -- |
|  | -20 | -14 | 4 | 3.2 | -- | -- | -- | -- |
|  | -20 | -26 | 4 | 3.2 | -- | -- | -- | -- |
|  | -14 | -16 | -2 | 3.2 | -- | -- | -- | -- |
|  | ***Left inferior frontal cluster*** k = 528, *p*_FWER-corr_ = .024 | | | |  | | | |
| Inferior frontal gyrus, pars triangularis (45) | -46 | 36 | 22 | 5.2* | -- | -- | -- | -- |
|  | ***Left superior frontal cluster*** k = 1189, *p*_FWER-corr_ = .001 | | | |  | | | |
| Superior frontal gyrus (6) | -24 | -6 | 60 | 6.1* | -- | -- | -- | -- |
|  |  | | | | ***Right inferior frontal and parieto-temporal cluster*** k = 6626, *p*_FWER-corr_ < .001 | | | |
| Inferior frontal gyrus, pars orbitalis (47) | -- | -- | -- | -- | 32 | 26 | -4 | 4.4* |
| Inferior frontal gyrus, pars opercularis (44) | -- | -- | -- | -- | 52 | 10 | 26 | 5.1* |
|  | -- | -- | -- | -- | 50 | 6 | 26 | 5.1* |
|  | -- | -- | -- | -- | 40 | 12 | 26 | 4.7* |
| Precentral gyrus (6/44) | -- | -- | -- | -- | 54 | 8 | 42 | 5.0* |
|  | -- | -- | -- | -- | 54 | 10 | 32 | 5.0* |
| *Continues in the next page* | -- | -- | -- | -- | 56 | 10 | 36 | 4.9* |
| *Continues from the previous page* | **Left Hemisphere** | | | | **Right Hemisphere** | | | |
| **Brain area (Brodmann area)** | **X** | **Y** | **X** | **Z-score** | **X** | **Y** | **X** | **Z-score** |
| Insula (47) | -- | -- | -- | -- | 32 | 28 | 0 | 4.4* |
| Inferior parietal lobule (40) | -- | -- | -- | -- | 34 | -44 | 46 | 4.1 |
|  | -- | -- | -- | -- | 38 | -40 | 42 | 4.1 |
| Supramarginal gyrus (2/3/40) | -- | -- | -- | -- | 60 | -20 | 26 | 4.8* |
|  | -- | -- | -- | -- | 62 | -30 | 24 | 4.6* |
|  | -- | -- | -- | -- | 42 | -38 | 40 | 4.2 |
| Superior temporal gyrus (21/22) | -- | -- | -- | -- | 56 | -2 | -2 | 4.8* |
|  | -- | -- | -- | -- | 52 | 2 | -8 | 4.7* |
|  | -- | -- | -- | -- | 46 | -30 | 4 | 4.1 |
|  | ***Bilateral parieto-occipital cluster*** k = 6103, *p*_FWER-corr_ < .001 | | | | | | | |
| Precentral gyrus (6) | -52 | -2 | 46 | 3.5 | -- | -- | -- | -- |
|  | -52 | 4 | 46 | 3.4 | -- | -- | -- | -- |
| Postcentral gyrus (4) | -60 | -20 | 26 | 3.9 | -- | -- | -- | -- |
|  | -54 | -20 | 32 | 3.8 | -- | -- | -- | -- |
|  | -48 | -18 | 44 | 3.6 | -- | -- | -- | -- |
| Superior parietal lobule (7) | -28 | -48 | 70 | 3.4 | 24 | -50 | 70 | 3.6 |
|  | -32 | -48 | 68 | 3.3 | -- | -- | -- | -- |
| Inferior parietal lobule (2/7/40) | -44 | -40 | 46 | 5.8* | -- | -- | -- | -- |
|  | -52 | -34 | 50 | 5.5* | -- | -- | -- | -- |
|  | -28 | -56 | 42 | 5.4* | -- | -- | -- | -- |
| Supramarginal gyrus (40) | -58 | -32 | 38 | 4.6* | -- | -- | -- | -- |
|  | -56 | -38 | 24 | 3.8 | -- | -- | -- | -- |
| Precuneus (7) | -10 | -70 | 48 | 5.7* | 14 | -66 | 50 | 5.4* |
| Middle occipital gyrus (7) | -26 | -64 | 40 | 5.2* | -- | -- | -- | -- |
|  | ***Bilateral inferior temporo-cerebellar cluster*** k = 6873, *p*_FWER-corr_ < .001 | | | | | | | |
| Inferior temporal gyrus (20) | -48 | -42 | -16 | 4.2 | 46 | -50 | -12 | 4.3 |
|  | -- | -- | -- | -- | 48 | -46 | -12 | 4.2 |
| Fusiform gyrus (18/19/37) | -26 | -72 | -8 | 4.8* | 30 | -72 | -10 | 4.4 |
|  | -40 | -50 | -10 | 4.8* | -- | -- | -- | -- |
|  | -36 | -62 | -6 | 4.5* | -- | -- | -- | -- |
| Lingual gyrus (18) | -- | -- | -- | -- | 20 | -76 | -6 | 4.5* |
|  | -- | -- | -- | -- | 20 | -72 | -8 | 4.4* |
| Cerebellum | -8 | -74 | -22 | 4.5* | 32 | -62 | -22 | 4.6* |
|  | -32 | -58 | -24 | 4.5* | -- | -- | -- | -- |
|  | -34 | -66 | -26 | 4.4* | -- | -- | -- | -- |
|  | -36 | -56 | -22 | 4.1 | -- | -- | -- | -- |
|  | -38 | -60 | -22 | 4.1 | -- | -- | -- | -- |
| Vermis | -- | -- | -- | -- | 6 | -76 | -22 | 4.7* |

# Table S3. The table reports the significant brain activations in the Human > Non-Human contrast (i.e., the main effect of Co-actor emerged from the ANOVA). All the reported effects meet a family-wise error rate (FWER) correction at the cluster level. (*) indicates peaks also surviving an FWER correction at the voxel level. We report a maximum of 16 coordinates (local maxima) per cluster, each placed at least 4 mm apart, as reported by default in SPM12. k = number of voxels in a given cluster. X’, ‘Y’, and ‘Z’ refer to MNI stereotaxic coordinates.

|  | **Left Hemisphere** | | | | **Right Hemisphere** | | | |
| --- | --- | --- | --- | --- | --- | --- | --- | --- |
| **Brain area (Brodmann area)** | **X** | **Y** | **Z** | **Z-score** | **X** | **Y** | **Z** | **Z-score** |
|  | ***Left middle temporal cluster*** k = 593, *p*_FWER-corr_ = .017 | | | | ***Right middle temporal cluster*** k = 730, *p*_FWER-corr_ = .008 | | | |
| Middle temporal gyrus (21/37) | -54 | -70 | 8 | 5.3* | 52 | -66 | 4 | 5.7* |
|  | -- | -- | -- | -- | 60 | -46 | -2 | 3.8 |

# Table S4. The table reports the significant brain activations in the regression analyses, indicating the brain areas which activity was predicted by the strength of the individual observation-related post-error slowing (oPES) effect, in the two Goal conditions separately. No interaction with the factor Co-actor was found, except for one occipital cluster (k = 455, *p_FWER-corr_*=.032) having its local maxima at (-38 -78 -2) which was more associated with the oPES generated in the Non-Human than Human condition in the Individual goal condition. In bold, the brain coordinates included in the main effect Error > NoError from the ANOVA (see Fig. 4 in the man text). All the reported effects meet an FWER correction at the cluster-level. We report in the table a maximum of 16 coordinates (local maxima) per cluster, each placed at least 4 mm apart, as reported by default in SPM12. k = number of voxels in a given cluster. ‘pMFC’ = posterior part of the medial frontal cortex; ‘pre-SMA’ = pre-supplementary motor area. ‘X’, ‘Y’, and ‘Z’ refer to MNI stereotaxic coordinates.

|  | **Left Hemisphere** | | | | **Right Hemisphere** | | | |
| --- | --- | --- | --- | --- | --- | --- | --- | --- |
| **Brain area (Brodmann area)** | **X** | **Y** | **Z** | **Z-score** | **X** | **Y** | **Z** | **Z-score** |
| *(a) Shared goal condition* | ***Posterior medio-frontal cluster*** k = 962, *p*_FWER-corr_ < .001 | | | | | | | |
| Middle cingulum (24) | -- | -- | -- | -- | 4 | 26 | 36 | 3.3 |
| pMFC – pre-SMA – Middle cingulum (6/32) | -- | -- | -- | -- | **0** | **16** | **46** | **3.9** |
|  | -- | -- | -- | -- | **2** | **16** | **56** | **3.7** |
| Superior frontal gyrus (6) | -- | -- | -- | -- | 18 | 8 | 56 | 3.9 |
| SMA (6) | -- | -- | -- | -- | 10 | 8 | 56 | 3.5 |
|  |  | | | | ***Right inferior frontal and insular cluster*** k = 670, *p*_FWER-corr_ = .003 | | | |
| Inferior frontal gyrus, pars triangularis (45) | -- | -- | -- | -- | 48 | 34 | 6 | 3.1 |
| Inferior frontal gyrus, pars orbitalis (47) | -- | -- | -- | -- | **54** | **30** | **-2** | **3.4** |
| Insula | -- | -- | -- | -- | 36 | 16 | 2 | 4.0 |
| Superior temporal pole | -- | -- | -- | -- | 52 | 12 | -6 | 4.5 |
|  |  | | | | ***Right occipito-temporal cluster*** k = 577, *p*_FWER-corr_ = .007 | | | |
| Middle temporal gyrus (37) | -- | -- | -- | -- | 52 | -70 | 4 | 4.1 |
| Middle occipital gyrus (18, 19, 37) | -- | -- | -- | -- | 44 | -74 | 10 | 3.7 |
|  | -- | -- | -- | -- | 32 | -84 | 14 | 3.7 |
|  | -- | -- | -- | -- | 36 | -86 | 12 | 3.7 |
|  | -- | -- | -- | -- | 26 | -84 | 12 | 3.6 |
| Inferior occipital gyrus (19) | -- | -- | -- | -- | 38 | -88 | -4 | 3.6 |
| *(b) Individual goal condition* |  | | | | ***Right posterior parietal cluster*** k = 1873, *p*_FWER-corr_ < .001 | | | |
| Superior parietal gyrus (7) | -- | -- | -- | -- | 16 | -68 | 60 | 3.8 |
| Supramarginal gyrus (40) |  |  |  |  | 58 | -42 | 30 | 3.9 |
| Angular gyrus (7/40) | -- | -- | -- | -- | **34** | **-58** | **36** | **4.3** |
|  | -- | -- | -- | -- | **36** | **-62** | **38** | **4.1** |
|  | -- | -- | -- | -- | **38** | **-68** | **40** | **4.0** |
|  | -- | -- | -- | -- | **38** | **-56** | **54** | **3.5** |
|  | -- | -- | -- | -- | 40 | -76 | 26 | 4.1 |
| *Continues in the next page* | -- | -- | -- | -- | 48 | -48 | 28 | 3.4 |
| *Continues from the previous page* | **Left Hemisphere** | | | | **Right Hemisphere** | | | |
| **Brain area (Brodmann area)** | **X** | **Y** | **Z** | **Z-score** | **X** | **Y** | **Z** | **Z-score** |
|  | -- | -- | -- | -- | 56 | -52 | 34 | 3.3 |
| Precuneus (7) | -- | -- | -- | -- | 2 | -64 | 60 | 3.4 |
|  | -- | -- | -- | -- | 20 | -56 | 40 | 3.2 |
| Superior temporal gyrus (41) | -- | -- | -- | -- | 42 | -44 | 16 | 3.6 |
| Middle temporal gyrus (21) | -- | -- | -- | -- | 48 | -46 | 16 | 3.6 |
| Middle occipital gyrus (19) | -- | -- | -- | -- | 32 | -80 | 32 | 3.5 |
|  | **White matter cluster** k = 424, *p*_FWER-corr_ = .039 | | | | | | | |
| Corpus callosum | -10 | -12 | 34 | 3.7 | 8 | -18 | 22 | 4.3 |
|  | -- | -- | -- | -- | 8 | -18 | 32 | 3.6 |
| Superior corona radiata | -22 | -10 | 28 | 3.8 | -- | -- | -- | -- |
|  | ***Left occipito-temporal cluster*** k = 1561, *p*_FWER-corr_ < .001 | | | |  |  |  |  |
| Middle temporal gyrus | -44 | -60 | 4 | 4.1 | -- | -- | -- | -- |
| Inferior temporal gyrus (37) | -50 | -58 | -14 | 3.8 | -- | -- | -- | -- |
|  | -46 | -52 | -6 | 3.5 | -- | -- | -- | -- |
| Fusiform gyrus (19, 37) | -48 | -54 | -18 | 3.9 | -- | -- | -- | -- |
|  | -36 | -48 | -22 | 3.6 | -- | -- | -- | -- |
|  | -32 | -78 | -16 | 3.4 | -- | -- | -- | -- |
|  | -36 | -76 | -16 | 3.3 | -- | -- | -- | -- |
|  | -40 | -42 | -20 | 3.3 | -- | -- | -- | -- |
| Middle occipital gyrus (18, 19) | -28 | -78 | 8 | 3.9 | -- | -- | -- | -- |
|  | -22 | -82 | 4 | 3.8 | -- | -- | -- | -- |
|  | -34 | -78 | 26 | 3.5 | -- | -- | -- | -- |
|  | -28 | -64 | -16 | 3.5 | -- | -- | -- | -- |
|  | -30 | -70 | 8 | 3.4 | -- | -- | -- | -- |
|  | -42 | -70 | -22 | 3.2 | -- | -- | -- | -- |
|  | -32 | -66 | 8 | 3.1 | -- | -- | -- | -- |
| Inferior occipital gyrus (19) | -32 | -78 | -8 | 3.2 | -- | -- | -- | -- |

# Video S1 (separate file). Experimental task overview. Here, we show an example of one trial for the Human and Non-Human co-actors. For the sake of clarity, the event succession is slowed down with respect to the actual trial timeline. Importantly, although, in the video, the participant’s response is represented at the bottom right of the screen, during the task, participants did not actually see their own hand.

# Video S2 (separate file). Example trials. For illustrative purposes, we show only one target combination of notes, that is, C – G (signaled by the pink melody in the Shared goal condition, and the light blue – red notes in the Individual goal condition), for each of the eight possible combinations of the factors Goal condition, Co-actor, and Trial-type. In the Error trials, the co-actor, either human or non-human, plays the wrong note, that is, plays G instead of C. Importantly, although, in the video, the participant’s response is represented at the bottom right of the screen, during the task, participants did not actually see their own hand.

**References**

Barr, D. J., Levy, R., Scheepers, C., & Tily, H. J. (2013). Random effects structure for confirmatory hypothesis testing: Keep it maximal. *Journal of Memory and Language*, *68*(3), 255–278. https://doi.org/10.1016/j.jml.2012.11.001

Bates, D., Mächler, M., Bolker, B., & Walker, S. (2015). Fitting Linear Mixed-Effects Models Using lme4. *Journal of Statistical Software*, *67*(1). https://doi.org/10.18637/jss.v067.i01

Chang, C.-C., & Lin, C.-J. (2011). LIBSVM. *ACM Transactions on Intelligent Systems and Technology*, *2*(3), 1–27. https://doi.org/10.1145/1961189.1961199

Flandin, G., & Friston, K. J. (2019). Analysis of family‐wise error rates in statistical parametric mapping using random field theory. *Human Brain Mapping*, *40*(7), 2052–2054. https://doi.org/10.1002/hbm.23839

Fox, J., & Weisberg, S. (2018). *An R Companion to Applied Regression* (Third Edition). SAGE Publications.

Gallucci, M. (2019). *GAMLj: General analyses for linear models. [jamovi module]. Retrieved from https://gamlj.github.io/.*

Gardumi, A., Ivanov, D., Hausfeld, L., Valente, G., Formisano, E., & Uludağ, K. (2016). The effect of spatial resolution on decoding accuracy in fMRI multivariate pattern analysis. *NeuroImage*, *132*, 32–42. https://doi.org/10.1016/j.neuroimage.2016.02.033

JASP Team. (2018). *JASP. [Computer software]. Retrieved from https://jasp-stats.org.*

Kumle, L., Võ, M. L.-H., & Draschkow, D. (2018). *Mixedpower: a library for estimating simulation-  based power for mixed models in R.* Zenodo. https://doi.org/10.5281/zenodo.1341048

Kumle, L., Võ, M. L.-H., & Draschkow, D. (2021). Estimating power in (generalized) linear mixed models: An open introduction and tutorial in R. *Behavior Research Methods*, *53*(6), 2528–2543. https://doi.org/10.3758/s13428-021-01546-0

Kuznetsova, A., Brockhoff, P. B., & Christensen, R. H. B. (2017). lmerTest Package: Tests in Linear Mixed Effects Models. *Journal of Statistical Software*, *82*(13). https://doi.org/10.18637/jss.v082.i13

Lakens, D., Scheel, A. M., & Isager, P. M. (2018). Equivalence Testing for Psychological Research: A Tutorial. *Advances in Methods and Practices in Psychological Science*, *1*(2), 259–269. https://doi.org/10.1177/2515245918770963

Lenth, R. (2024). *emmeans: Estimated Marginal Means, aka Least-Squares Means. R package version 1.10.0*.

Lüdecke, D., Ben-Shachar, M., Patil, I., Waggoner, P., & Makowski, D. (2021). performance: An R Package for Assessment, Comparison and Testing of Statistical Models. *Journal of Open Source Software*, *6*(60), 3139. https://doi.org/10.21105/joss.03139

Morey, R. D., & Rouder, J. N. (2018). *BayesFactor: Computation of Bayes Factors for Common Designs. [R package]. Retrieved from https://cran.r-project.org/package=BayesFactor.*

Rouder, J. N., Speckman, P. L., Sun, D., Morey, R. D., & Iverson, G. (2009). Bayesian t tests for accepting and rejecting the null hypothesis. *Psychonomic Bulletin & Review*, *16*(2), 225–237. https://doi.org/10.3758/PBR.16.2.225

Sacheli, L. M., Musco, M. A., Zazzera, E., Banfi, G., & Paulesu, E. (2022). How shared goals shape action monitoring. *Cerebral Cortex*, 1–18. https://doi.org/10.1093/cercor/bhac019

Sacheli, L. M., Musco, M. A., Zazzera, E., & Paulesu, E. (2021). Mechanisms for mutual support in motor interactions. *Scientific Reports*, *11*(1), 1–16. https://doi.org/10.1038/s41598-021-82138-y

Scandola, M., & Tidoni, E. (2024). Reliability and Feasibility of Linear Mixed Models in Fully Crossed Experimental Designs. *Advances in Methods and Practices in Psychological Science*, *7*(1). https://doi.org/10.1177/25152459231214454

Schrouff, J., Rosa, M. J., Rondina, J. M., Marquand, A. F., Chu, C., Ashburner, J., Phillips, C., Richiardi, J., & Mourão-Miranda, J. (2013). PRoNTo: Pattern Recognition for Neuroimaging Toolbox. *Neuroinformatics*, *11*(3), 319–337. https://doi.org/10.1007/s12021-013-9178-1

The jamovi project. (2022). *jamovi. Retrieved from https://www.jamovi.org.*

Worsley, K. J., & Friston, K. J. (1995). Analysis of fMRI Time-Series Revisited—Again. *NeuroImage*, *2*(3), 173–181. https://doi.org/10.1006/nimg.1995.1023

1. R syntax of the most complete, non-singular, converging model:
   glmer(ACC ~ 1+(1|Participant) + (1|Participant:GoalCondition) +

   (1|Participant:GoalCondition:Co-actor) + (1|Participant:GoalCondition:Trial-type) +

   GoalCondition * Co-actor * Trial-type, data = dt, family = binomial (link="logit"),

   control = glmerControl(optimizer = "bobyqa")) [↑](#footnote-ref-1)
2. R syntax of the most complete, non-singular, converging model:

   lmer(RT ~ 1+(1|Participant) +

   (1|Participant:GoalCondition) + (1|Participant:Co-Actor) + (1|Participant:Trial-type) +

   (1|Participant:GoalCondition:Co-Actor) + (1|Participant:GoalCondition:Trial-type) + (1|Participant:Co-Actor:Trial-type) +

   (1|Participant:GoalCondition:Co-Actor:Trial-type) +

   GoalCondition*Co-Actor*Trial-type, data = dt) [↑](#footnote-ref-2)
